# Brain fractalkine-CX3CR1 signalling is anti-obesity system as anorexigenic and anti-inflammatory actions in diet-induced obese mice

**DOI:** 10.1038/s41598-022-16944-3

**Published:** 2022-07-23

**Authors:** Namiko Kawamura, Goro Katsuura, Nobuko Yamada-Goto, Riho Nakama, Yuki Kambe, Atsuro Miyata, Tomoyuki Furuyashiki, Shuh Narumiya, Yoshihiro Ogawa, Akio Inui

**Affiliations:** 1 Department of Medicine and Bioregulatory Science, Graduate School of Medical Sciences, Kyushu University, Fukuoka, Japan; 2 Drug Discovery of Next-Generation GcMAF, Kagoshima University Graduate School of Medical and Dental Sciences, Kagoshima, Japan; 3 Health Center, Keio University, Tokyo, Japan; 4 Division of Endocrinology, Metabolism and Nephrology, Department of Internal Medicine, Keio University School of Medicine, Tokyo, Japan; 5 Department of Pharmacology, Kagoshima University Graduate School of Medical and Dental Sciences, Kagoshima, Japan; 6 Division of Pharmacology, Graduate School of Medicine, Kobe University, Hyogo, Japan; 7 Department of Drug Discovery Medicine, Graduate School of Medicine, Kyoto University, Kyoto, Japan; 8 Pharmacological Department of Herbal Medicine, Kagoshima University Graduate School of Medical and Dental Sciences, Kagoshima, Japan

**Keywords:** Neuroscience, Diseases

## Abstract

Fractalkine is one of the CX3C chemokine family, and it is widely expressed in the brain including the hypothalamus. In the brain, fractalkine is expressed in neurons and binds to a CX3C chemokine receptor 1 (CX3CR1) in microglia. The hypothalamus regulates energy homeostasis of which dysregulation is associated with obesity. Therefore, we examined whether fractalkine-CX3CR1 signalling involved in regulating food intake and hypothalamic inflammation associated with obesity pathogenesis. In the present study, fractalkine significantly reduced food intake induced by several experimental stimuli and significantly increased brain-derived neurotrophic factor (BDNF) mRNA expression in the hypothalamus. Moreover, tyrosine receptor kinase B (TrkB) antagonist impaired fractalkine-induced anorexigenic actions. In addition, compared with wild-type mice, CX3CR1-deficient mice showed a significant increase in food intake and a significant decrease in BDNF mRNA expression in the hypothalamus. Mice fed a high-fat diet (HFD) for 16 weeks showed hypothalamic inflammation and reduced fractalkine mRNA expression in the hypothalamus. Intracerebroventricular administration of fractalkine significantly suppressed HFD-induced hypothalamic inflammation in mice. HFD intake for 4 weeks caused hypothalamic inflammation in CX3CR1-deficient mice, but not in wild-type mice. These findings suggest that fractalkine-CX3CR1 signalling induces anorexigenic actions via activation of the BDNF-TrkB pathway and suppresses HFD-induced hypothalamic inflammation in mice.

## Introduction

Fractalkine is a member of the CX3C chemokine family, and it is widely expressed in brain regions such as the hypothalamus, hippocampus, and cortex^1,2^. In the hypothalamus, fractalkine is expressed in the paraventricular nucleus (PVN) and lateral hypothalamus (LH)^1,3^. Fractalkine has a one-to-one relationship with the G protein-coupled receptor CX3C chemokine receptor 1 (CX3CR1) which is expressed throughout the whole brain almost uniformly^2^. In the brain, fractalkine is expressed in neurons whereas CX3CR1 is expressed exclusively in microglia^2^. Fractalkine-CX3CR1 signalling regulates various functions of the central nervous system (CNS), including immune responses, stress responses, pain, and cognition^4–6^.

The CNS plays a key role in sensing and controlling the energy status of the organism^7^, and the hypothalamus is one of the most important brain regions involved in the central control of feeding and energy expenditure. The hypothalamus is comprised of two neuronal subpopulations, the orexigenic neuropeptide Y (NPY)/agouti-related peptide (AgRP) and anorexigenic proopiomelanocortin (POMC)/cocaine- and amphetamine-regulated transcript (CART) neurons^8^, and food intake under physiological and pathophysiological states is regulated by complex and integrated control of these neurons. Furthermore, brain-derived neurotrophic factor (BDNF) is a key molecule involved in the regulation of food intake^9–12^. BDNF is widely distributed in the brain^13^. In the hypothalamus, BDNF is highly expressed in the ventromedial nucleus (VMH) and moderately in the PVN and LH^9^. The BDNF receptor tyrosine receptor kinase B (TrkB) is distributed in the arcuate nucleus (ARC), PVN, LH, VMH, and dorsomedial nucleus of the hypothalamus^14^. Recent studies reported that intracerebroventricular administration of BDNF decreases food intake^10–12^, and chronic ventricular infusion of BDNF reverses hyperphagia in BDNF heterozygous animals^9^. A previous study reported that intracerebroventricular administration of fractalkine increases BDNF in the hippocampus^15^.

Obesity is defined as an increased adipose mass resulting from chronic excess energy intake over energy expenditure, and is associated with a higher risk of lifestyle-related cardiovascular and metabolic disorders, such as hypertension, diabetes, and hyperlipidemia. Dietary long-chain saturated fatty acids are the main triggers of hypothalamic inflammation in obesity^16^. Experimental activation of hypothalamic inflammatory pathways induces hyperphagia and weight gain, predisposes to diet-induced obesity and blunts the anorexigenic effects of insulin and leptin^17^. Conversely, reduction of hypothalamic inflammation can reduce food intake and body weight and improve hypothalamic insulin and leptin sensitivity^17,18^. Recent evidence demonstrated that fractalkine suppresses high-fat diet (HFD) feeding and hypothalamic microglial activation induced by consumption of a HFD^19^. Furthermore, it has been reported that female CX3CR1-deficient mice fed a HFD show an increase in mRNA expression of hypothalamic inflammatory markers compared with wild-type mice^19^.

In the present study, we examined the effects of fractalkine on food intake in mice under several experimental conditions. In addition, to explore the possible mechanisms underlying the fractalkine-related regulation of food intake, we particularly examined the involvement of BDNF in the actions of fractalkine. Moreover, we assessed the contribution of fractalkine-CX3CR1 signalling to HFD-induced hypothalamic inflammation in mice.

## Results

### Effects of fasting-refeeding on the mRNA expression of fractalkine, CX3CR1, and feeding regulatory peptides and their receptors in the hypothalamus

**Figure 1:**
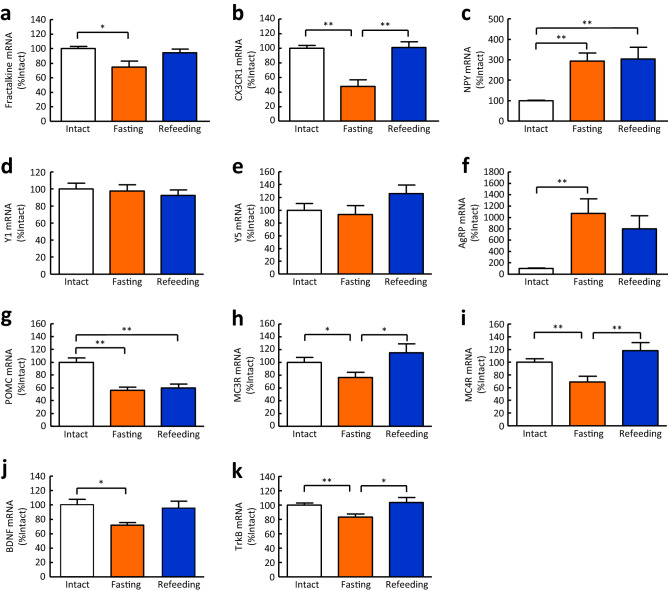
Effects of fasting-refeeding on mRNA expression of fractalkine, CX3CR1, and feeding regulatory peptides and their receptors in the hypothalamus of mice. (**a**) Fractalkine, (**b**) CX3CR1, (**c**) NPY, (**d**) Y1 receptor, (**e**) Y5 receptor, (**f**) AgRP, (**g**) POMC, (**h**) MC3R, (**i**) MC4R, (**j**) BDNF, and (**k**) TrkB were observed in the hypothalamus of mice after 48-h fasting and 4-h refeeding. Results are expressed as mean ± SEM for 6–13 mice. Significant differences: *p < 0.05, **p < 0.01.

The mRNA expression of fractalkine and its receptor CX3CR1 was significantly decreased after 48-h fasting, and returned to intact levels after 4-h refeeding (Fig. 1a, b). NPY mRNA expression was significantly increased after 48-h fasting (Fig. 1c), whereas the mRNA expression of NPY Y1 and Y5 receptors was not changed by 48-h fasting (Fig. 1d, e). AgRP mRNA expression was significantly increased after 48-h fasting (Fig. 1f). On the other hand, POMC mRNA expression was significantly decreased after 48-h fasting and after 4-h refeeding (Fig. 1g). The mRNA expression of melanocortin-3 receptor (MC3R) and melanocortin-4 receptor (MC4R) was significantly decreased after 48-h fasting, and returned to intact levels after 4-h refeeding (Fig. 1h, i). BDNF mRNA expression was significantly decreased after 48-h fasting and returned to intact levels after 4-h refeeding (Fig. 1j). TrkB mRNA expression was also significantly decreased after 48-h fasting and returned to intact levels after 4-h refeeding (Fig. 1k).

### Effects of intracerebroventricular administration of fractalkine on food intake in mice

**Figure 2:**
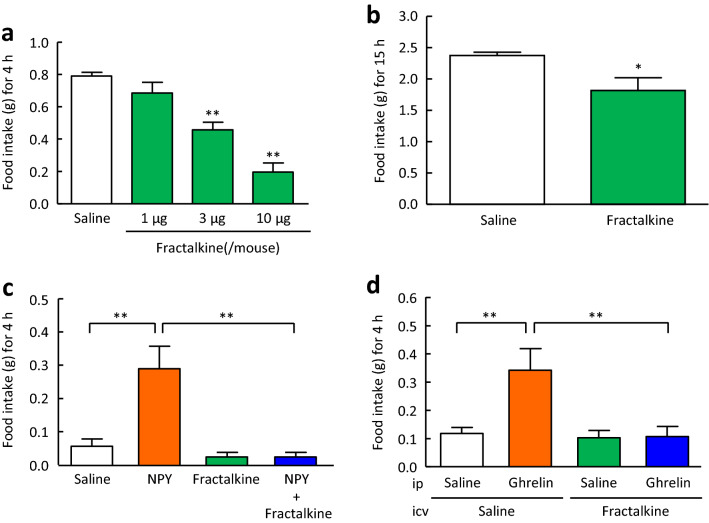
Effects of intracerebroventricular administration of fractalkine on food intake in mice. (**a**) Effects of intracerebroventricular administration of fractalkine (1, 3, and 10 μg/mouse) on food intake during 4-h refeeding after 48-h fasting in mice. (**b**) Effects of intracerebroventricular administration of fractalkine (3 μg/mouse) on nocturnal food intake in mice. (**c**) Effects of intracerebroventricular administration of fractalkine (3 μg/mouse) on NPY-induced (20 μg/mouse, intracerebroventricular administration) food intake in mice. (**d**) Effects of intracerebroventricular administration of fractalkine (3 μg/mouse) on ghrelin-induced (360 μg/kg, intraperitoneal administration) food intake in mice. Results are expressed as mean ± SEM for 5–12 mice. Significant differences: *p < 0.05, **p < 0.01.

Food intake during 4-h refeeding after 48-h fasting was significantly suppressed by intracerebroventricular administration of fractalkine in a dose-dependent manner (3 and 10 μg/mouse) compared with saline treatment (Fig. 2a). Intracerebroventricular administration of fractalkine (3 μg/mouse) 1 h before the start of the dark phase significantly suppressed nocturnal food intake compared with saline treatment (Fig. 2b). Compared with saline treatment, concomitant intracerebroventricular administration of fractalkine (3 μg/mouse) with NPY (20 μg/mouse) significantly suppressed the food intake induced by NPY (Fig. 2c). In addition, intracerebroventricular administration of fractalkine (3 μg/mouse) significantly suppressed food intake induced by intraperitoneal administration of ghrelin (360 μg/kg) (Fig. 2d).

### Involvement of BDNF in the anorexigenic effect of fractalkine in mice

**Figure 3:**
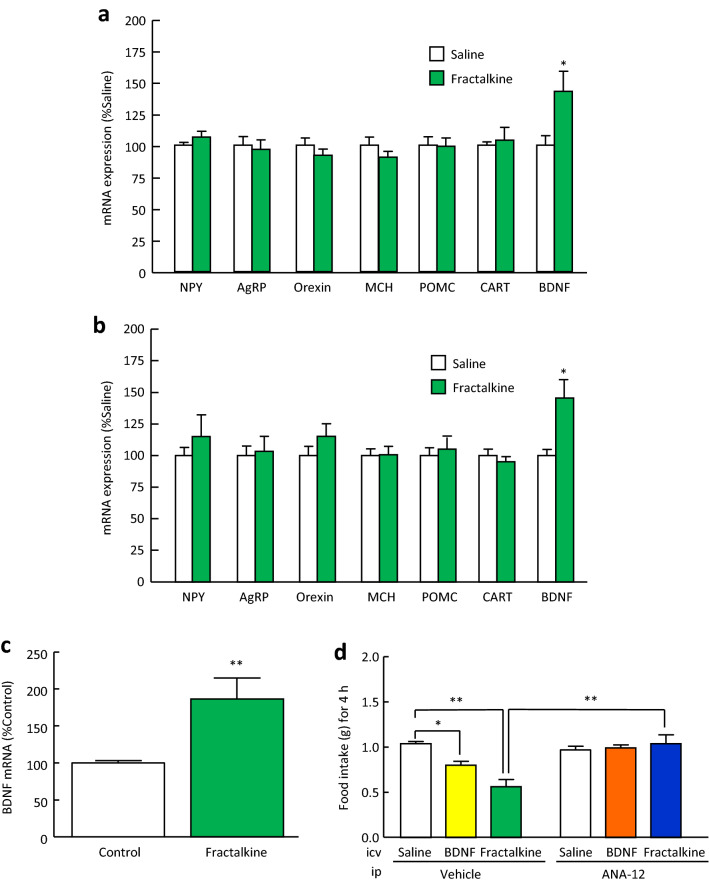
Involvement of BDNF in the anorexigenic effect of fractalkine in mice. (**a**) mRNA expression in the hypothalamus of normal mice was examined at 4 h after intracerebroventricular administration of fractalkine (3 μg/mouse). (**b**) mRNA expression in the hypothalamus of refed mice was examined at 4 h after intracerebroventricular administration of fractalkine. Fractalkine (3 μg/mouse) was intracerebroventricularly administered before 4-h refeeding after 48-h fasting. (**c**) BDNF mRNA expression in MG6 cells was examined after application of fractalkine (100 nM) for 3 h. (**d**) TrkB antagonist ANA-12 (0.5 mg/kg) was intraperitoneally administered at 30 min before intracerebroventricular administration of fractalkine (3 μg/mouse) and BDNF (0.5 μg/mouse). Results are expressed as mean ± SEM for 6–14 mice. Significant differences: *p < 0.05, **p < 0.01.

Intracerebroventricular administration of fractalkine (3 μg/mouse) significantly increased BDNF mRNA expression in the hypothalamus of normal mice to 143% of saline treatment (Fig. 3a). In addition, intracerebroventricular administration of fractalkine (3 μg/mouse) significantly increased BDNF mRNA expression in the hypothalamus of mice after 4-h refeeding to 146% of saline treatment (Fig. 3b). On the other hand, intracerebroventricular administration of fractalkine (3 μg/mouse) did not change the mRNA expression of NPY, AgRP, orexin, melanin-concentrating hormone (MCH), POMC, or CART in the hypothalamus of normal or refed mice (Fig. 3a, b). Moreover, application of fractalkine (100 nM) for 3 h significantly increased BDNF mRNA expression in MG6 cells, a mouse microglia cell line (Fig. 3c). To examine the involvement of BDNF in the anorectic effect of fractalkine, the TrkB antagonist ANA-12 (0.5 mg/kg) was intraperitoneally administrated at 30 min before intracerebroventricular administration of fractalkine (3 μg/mouse). ANA-12 significantly attenuated the suppressive actions of fractalkine, as well as BDNF, on food intake during 4-h refeeding after 48-h fasting (Fig. 3d).

### Cumulative food intake and BDNF mRNA expression in the hypothalamus of CX3CR1-deficient mice

**Figure 4:**
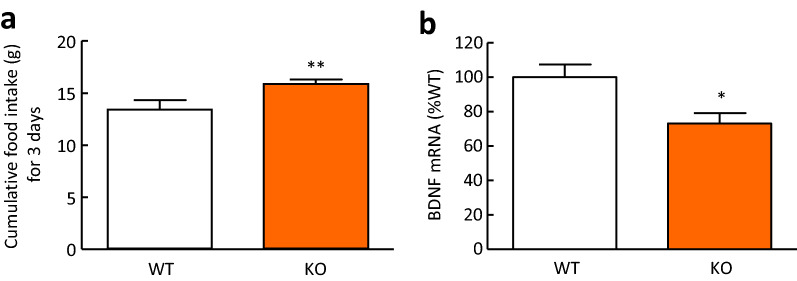
Cumulative food intake and BDNF mRNA expression in the hypothalamus of CX3CR1-deficient mice. (**a**) Cumulative food intake for 3 days. (**b**) BDNF mRNA expression in the hypothalamus. WT: wild-type mice, KO: CX3CR1-deficiet mice. Results are expressed as mean ± SEM for 7–8 mice. Significant differences: *p < 0.05, **p < 0.01.

To examine the precise involvement of fractalkine in food intake, we performed experiments using CX3CR1-deficient mice. Cumulative food intake for 3 days in CX3CR1-deficient mice was significantly increased compared with that in wild-type mice (Fig. 4a). Moreover, hypothalamic BDNF mRNA expression in CX3CR1-deficient mice was significantly decreased compared with that in wild-type mice (Fig. 4b).

### Changes in the mRNA expression of proinflammatory cytokines, fractalkine, and CX3CR1 in the hypothalamus of mice fed a HFD

**Figure 5:**
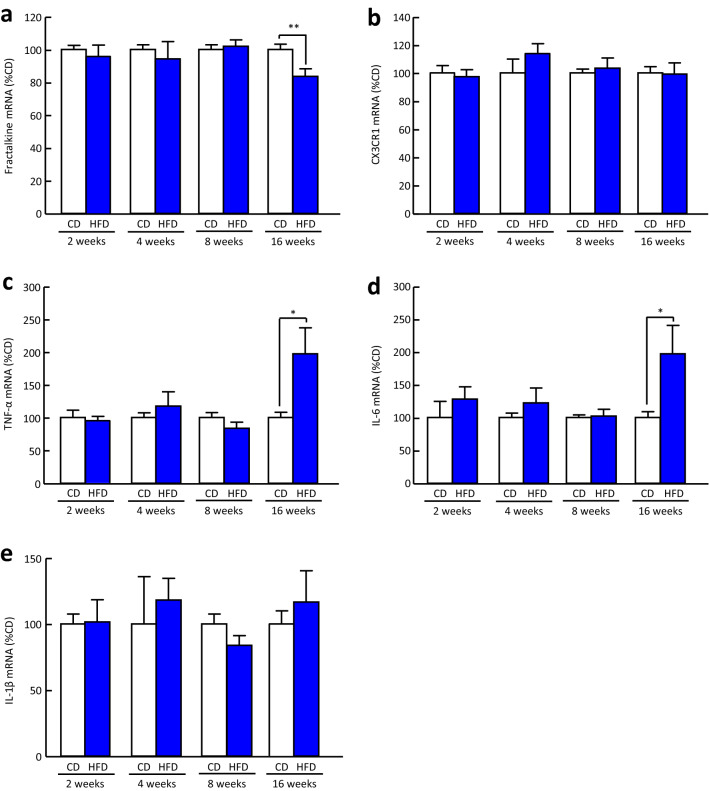
Changes in mRNA expression of fractalkine, CX3CR1, and proinflammatory cytokines in the hypothalamus of mice fed a HFD. (**a**) Fractalkine, (**b**) CX3CR1, (**c**) TNF-α, (**d**) IL-6, and (**e**) IL-1β mRNA expression in the hypothalamus of mice fed a HFD for 2, 4, 8, and 16 weeks. Results are expressed as mean ± SEM for 5–16 mice. Significant difference: *p < 0.05, **p < 0.01.

Hypothalamic fractalkine mRNA expression in mice fed a HFD for 16 weeks was significantly decreased to 84% of that in mice fed a control diet (CD), whereas its mRNA expression was not changed by HFD feeding for 2, 4, and 8 weeks (Fig. 5a). Hypothalamic CX3CR1 mRNA expression was not changed by HFD feeding for 2, 4, 8, and 16 weeks (Fig. 5b). The mRNA expression of tumour necrosis factor-α (TNF-α) and interleukin-6 (IL-6) in the hypothalamus of mice fed a HFD for 16 weeks, but not 2, 4, and 8 weeks, was significantly increased to 197 and 198% of that in mice fed a CD, respectively (Fig. 5c, d). On the other hand, interleukin-1β (IL-1β) mRNA expression in mice fed a HFD for 2, 4, 8, and 16 weeks was not different from that in mice fed a CD (Fig. 5e).

### Effects of intracerebroventricular administration of fractalkine on increased mRNA expression of TNF-α and IL-6 in the hypothalamus of mice fed a HFD

**Figure 6:**
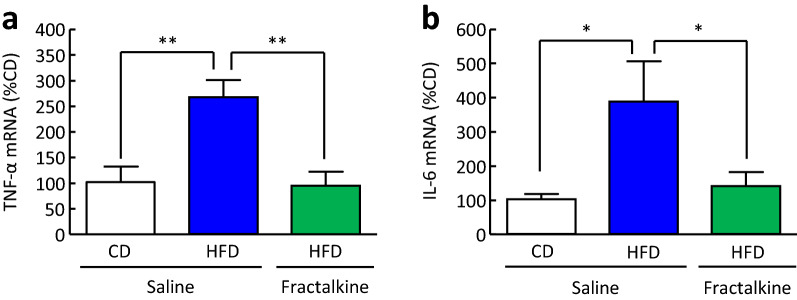
Effects of intracerebroventricular administration of fractalkine on mRNA expression of TNF-αand IL-6 in the hypothalamus of mice fed a HFD. (**a**) TNF-α and (**b**) IL-6 mRNA expression in the hypothalamus of mice fed a CD or HFD for 16 weeks at 3 h after intracerebroventricular administration of fractalkine. Results are expressed as mean ± SEM for 6–15 mice. Significant difference: *P < 0.05, **P < 0.01.

Fractalkine has anti-inflammatory actions^20–24^. Therefore, we examined the effects of intracerebroventricular administration of fractalkine on increased proinflammatory cytokine expression in the hypothalamus of mice fed a HFD for 16 weeks. The intracerebroventricular administration of fractalkine (3 μg/mouse) significantly decreased the increased mRNA expression of TNF-α and IL-6 in the hypothalamus of mice fed a HFD for 16 weeks by 65 and 64% as compared with saline-treated mice fed a HFD, respectively (Fig. 6a, b).

### Changes in the mRNA expression of TNF-α and IL-6 in the hypothalamus and cumulative food intake of CX3CR1-deficient mice fed a HFD

**Figure 7:**
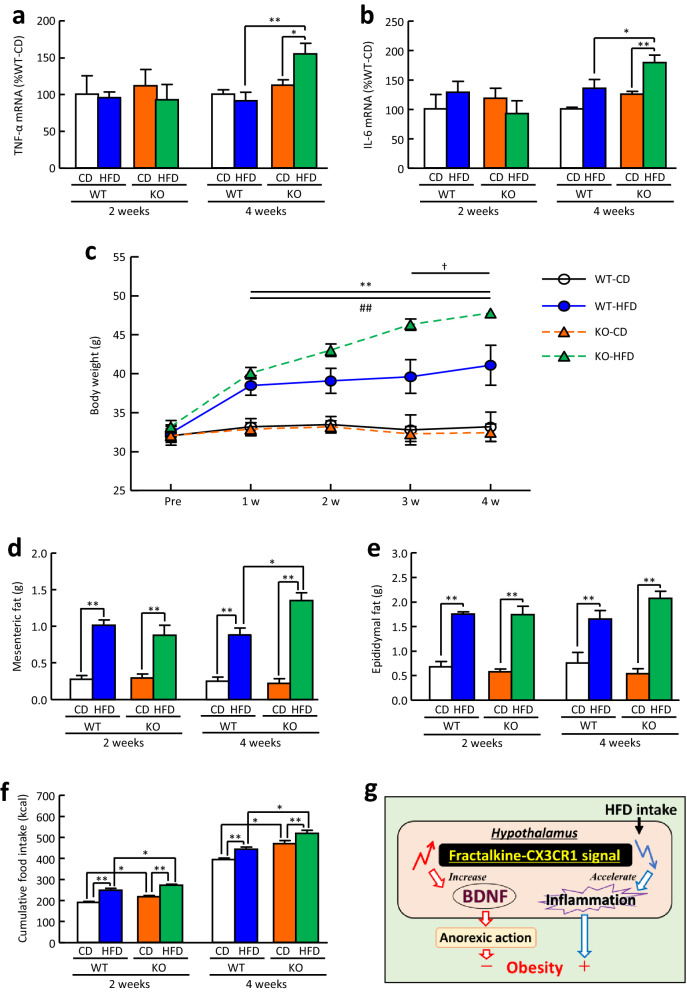
Changes in TNF-α and IL-6 mRNA expression in the hypothalamus and cumulative food intake and metabolic parameters of CX3CR1-deficient mice fed a HFD. (**a**) TNF-α and (**b**) IL-6 mRNA expression in the hypothalamus of CX3CR1-deficient mice fed a HFD for 2 and 4 weeks. (**c**) Body weight, (**d**) mesenteric fat, and (**e**) epididymal fat in CX3CR1-deficient mice fed a HFD for 2 and 4 weeks. (**f**) Cumulative food intake for 2 and 4 weeks in CX3CR1-deficient mice fed a HFD. (**g**) Proposed mechanisms of food intake regulation by fractalkine-CX3CR1 signalling. WT: wild-type mice, KO: CX3CR1-deficiet mice. Results are expressed as mean ± SEM for 4–11 mice. Significant difference in (**a**), (**b**), (**d**), (**e**), and (**f**): *p < 0.05, **p < 0.01. Significant difference in c: **p < 0.01; WT-HFD compared with WT-CD, ^##^*p* < 0.01; KO-HFD compared with KO-CD, ^†^*p* < 0.05; KO-HFD compared with WT-HFD.

In CX3CR1-deficient mice, HFD feeding for 4 weeks, but not 2 weeks, significantly increased TNF-α and IL-6 mRNA expression in the hypothalamus to 138% and 143%, respectively, compared with CD feeding, while wild-type mice did not (Fig. 7a, b). In the CD-feeding groups, TNF-α and IL-6 mRNA expression in the hypothalamus was not different between CX3CR1-deficient mice and wild-type mice (Fig. 7a, b). In both wild-type and CX3CR1-deficient mice, HFD feeding for more than a week induced a significant increase in body weight compared with CD feeding (Fig. 7c). Body weight in CX3CR1-deficient mice fed a HFD was significantly increased by HFD feeding for more than 3 weeks compared with that in wild-type mice fed a HFD (Fig. 7c). Mesenteric fat and epididymal fat were significantly increased by HFD feeding for more than 2 weeks in both wild-type and CX3CR1-deficient mice compared with that in CD feeding (Fig. 7d, e). CX3CR1-deficient mice fed a HFD for 4 weeks showed a significant increase in mesenteric fat, but not epididymal fat, compared with wild-type mice fed a HFD (Fig. 7d, e). Cumulative food intake of both the CD and HFD for 2 and 4 weeks in CX3CR1-deficient mice was significantly increased compared with that in wild-type mice (Fig. 7f).

## Discussion

The present study demonstrated that fractalkine suppresses food intake via activation of the BDNF-TrkB pathway and that reduction of fractalkine-CX3CR1 signalling exacerbates HFD-induced hypothalamic inflammation and accelerates obesity in HFD-fed mice (Fig. 7g).

The hypothalamus is considered an important region for the regulation of food intake. Consistent with previous report^25^, the present study showed that 48 h of fasting significantly increased orexigenic peptides and decreased anorexigenic factors in the hypothalamus. Fasting decreases plasma leptin, insulin, and glucose, which are involved in the regulation of gene expression in the hypothalamus. However, it is difficult to determine the independent role of each factor in the regulation of hypothalamic gene expression because leptin, insulin, and glucose tend to form regulatory loops with each other and co-vary. In the present study, we showed that mRNA expression of MC3R, MC4R, and TrkB, the receptor for BDNF, which acts as a downstream effector of MC4R signalling, is significantly reduced after 48 h of fasting and returns to intact levels after 4 h of refeeding. In mammals, MC3R and MC4R central signalling plays a major role in the regulation of appetite and metabolism. Neural circuits in the hypothalamus act to maintain body weight at a stable set point by activating behavioral, neuroendocrine, and autonomic pathways in response to acute changes in energy stores. MC4R is well known to act as a master regulator of body weight set point^26,27^. In contrast to the MC4R, deletion of the MC3R does not produce measurable hyperphagia or hypometabolism under normal conditions. However, MC3R regulates normal fasting response and adaptation to restricted feeding^28^. In the present study, fractalkine and CX3CR1 mRNA expression in the hypothalamus was altered after fasting-feeding. Therefore, we speculated that fractalkine-CX3CR1 signalling may act to modulate feeding.

In the present study, intracerebroventricular administration of fractalkine significantly reduced food intake during 4-h refeeding after 48-h fasting, nocturnal food intake, and food intake induced by NPY and ghrelin. A previous study showed that BDNF in the PVN inhibits deprivation-induced feeding, normal dark-phase feeding, and NPY-induced feeding^29^. Therefore, we considered the involvement of BDNF in the anorexigenic mechanisms of fractalkine. In the present study showed that intracerebroventricular administration of fractalkine significantly increased BDNF mRNA expression in the hypothalamus of mice. Moreover, the TrkB antagonist, ANA-12 significantly attenuated the suppressive actions of fractalkine on food intake during 4-h refeeding after 48-h fasting. The anorexigenic actions of BDNF are mediated by the high-affinity receptor TrkB, and mutations in BDNF or TrkB genes may account for certain types of obesity or other forms of eating disorders in humans^30^. Since there is little co-expression of TrkB and CART or NPY in the hypothalamic nucleus, it is unlikely that BDNF directly regulates POMC/CART or NPY/AgRP-expressing neurons in the ARC. However, there is some evidence for a link between melanocortin signalling and BDNF-TrkB signalling, suggesting that BDNF acts as a downstream effector of MC4R signalling to regulate energy balance^31^. BDNF is thought to interact with NPY to exert its anorexigenic effects^29^, but its direct action on NPY has not yet been established. Based on these findings and the present study, it is likely that BDNF acts downstream of the actions of these peptides and that fractalkine suppresses food intake via activation of BDNF-TrkB signalling without altering mRNA expression of other feeding regulatory peptides. Because CX3CR1 is only expressed on microglia, we used the microglia cell line MG6 cells to examine the effects of fractalkine on BDNF mRNA expression in an in vitro experiment. Application of fractalkine significantly increased BDNF mRNA expression in MG6 cells. However, BDNF is expressed in not only microglia but also neurons^32^. Stimulation of microglial cells by fractalkine induces an increase in extracellular adenosine levels^33^. Activation of the adenosine receptor A_2A_ receptor with the A_2A_ receptor agonist CGS21680 increases BDNF expression in developing rat primary cortical neurons^32^ and murine N9 microglial cells^34^. These reports^32–34^ suggest that the increase in hypothalamic BDNF mRNA expression induced by intracerebroventricular administration of fractalkine may be induced in neurons as well as microglia. Furthermore, we examined food intake and hypothalamic BDNF mRNA expression in CX3CR1-deficient mice. CX3CR1-deficient mice showed an increase in cumulative food intake for 3 days and a decrease in hypothalamic BDNF mRNA expression compared with wild-type mice. These findings suggest that fractalkine-CX3CR1 signalling has anorexigenic actions that are mediated by an increase in BDNF which is an anorexigenic peptide in the hypothalamus.

Obesity results from the dysregulation of energy metabolism^35^. In both human and animal models, diet-induced obesity is associated with increased circulating inflammatory markers such as TNF-α, IL-1β, and IL-6, which represent a key step in the development of insulin resistance in peripheral organs^36,37^. Fractalkine is widely present not only in the brain but also in peripheral tissues such as the heart, lung, kidney, muscle, testis, and adipose tissue^38^. CX3CR1-deficient mice exhibit glucose intolerance mainly due to beta cell dysfunction, while fractalkine treatment improves glucose tolerance and increases insulin secretion in wild-type mice^39^. Moreover, deficient fractalkine-CX3CR1 signalling exacerbates diet-induced insulin resistance, hepatic steatosis, and adipose tissue inflammation^40^. Furthermore, just as in peripheral tissues, in animal models of genetic and diet-induced obesity, inducing an inflammatory response in the hypothalamus leads to the molecular and functional resistance to the adipostatic hormones leptin and insulin, resulting in defective control of food intake and energy expenditure^17,41,42^. Experimental interventions that block hypothalamic inflammation reduce food intake, leptin resistance, and body weight in animals exposed to HFD feeding^16–18,43,44^. Depleting microglia from the hypothalamus of mice abolishes inflammation and neuronal stress induced by excess saturated fatty acid consumption^44^. In this context, these findings indicate that microglia are potently involved in brain inflammation induced by HFD feeding^44^. De Souza demonstrated that immune-related molecules, including proinflammatory cytokines, such as TNF-α, IL-1β, and IL-6, in the hypothalamus significantly increase after 16 weeks of HFD feeding^45^. Consistent with this finding, the present study showed that HFD feeding for 16 weeks, but not 2, 4, and 8 weeks, resulted in a significant increase in mRNA expression of TNF-α and IL-6 in the hypothalamus of mice.

Fractalkine levels are downregulated in the adipose tissue, plasma and brain in diet-induced obese mice^19,40,46^. The present study showed a significant decrease in the mRNA expression of fractalkine, but not CX3CR1, in the hypothalamus of mice fed a HFD for 16 weeks. Our previous report revealed that long-term HFD feeding in mice reduces fractalkine expression in the hippocampus and amygdala, which may be caused by decreases in plasma IGF-1 levels and brain BDNF levels induced by long-term HFD feeding^46^. Contrary to our results, previous study showed that HFD intake for 8 weeks increases fractalkine and CX3CR1 expression in the hypothalamus in male Swiss mice, whereas HFD intake does not change fractalkine and CX3CR1 expression in the hypothalamus of male Balb-c mice^47^. Moreover, another paper reported that male C57BL/6 J mice fed HFD for 18 weeks shows a decrease in fractalkine and CX3CR1, but not female^19^. These differences may relate to strain (Swiss mice are extremely DIO-sensitive) and sex.

Previous reports showed that exogenous fractalkine decreases TNF-α secretion induced by microglial activation^20^, while neutralization of endogenous brain fractalkine increases TNF-α production induced by lipopolysaccharide^22^. However, another report showed that inhibition of hypothalamic fractalkine reduces HFD-induced hypothalamic inflammation in male Swiss mice^47^. CX3CR1-deficient mice show excessive cellular activation and overproduction of inflammatory mediators, generally increasing susceptibility to CNS inflammatory diseases^48,49^. However, some studies have identified protective aspects of CX3CR1 deficiency^50^. These reports suggest that whether fractalkine promotes or protects inflammation depends on the state of inflammation and the conditions of the experiments, suggesting that the action of fractalkine-CX3CR1 signaling on inflammation is complexly regulated. In the present study, the intracerebroventricular administration of fractalkine significantly suppressed the increase in the mRNA expression of both TNF-α and IL-6 in the hypothalamus of mice fed a HFD for 16 weeks. Conversely, CX3CR1-deficient mice exhibited hypothalamic inflammation induced by HFD feeding for 4 weeks, whereas wild-type mice did not. Taken together, we hypothesized that reduced fractalkine-CX3CR1 signalling in the hypothalamus of mice fed a HFD may lead to exaggerated hypothalamic microglial activation and inflammatory responses induced by HFD consumption. In addition, body weight in CX3CR1-deficient mice fed a HFD was significantly increased by HFD feeding for more than 3 weeks compared with that in wild-type mice fed a HFD. However, contrary to our study, previous reports^40,51^ demonstrated that CX3CR1-deficient mice fed a HFD show similar changes in body weight to wild-type mice fed a HFD. A possible explanation for this discrepancy may reside in the several differences in the methodological procedures as follows; (1) in the present study, HFD feeding started at 14–16 weeks old, while HFD feeding started at 4 weeks old in previous study^40^, (2) in the present study, HFD containing 60% fat of total calories was used, while HFD containing 45% fat of total calories was used in previous study^51^. It seems likely that these methodological differences may induce the different results in each report. In the present study, CX3CR1-deficient mice fed a HFD for 4 weeks showed a significant increase in mesenteric fat as well as body weight compared with wild-type mice fed HFD, that is, CX3CR1-deficient mice may have increased body weight and mesenteric fat due to excess energy intake from increased HFD feeding. The CX3CR1-deficient mice used in the present study were systemically deficient but may reflect, at least in part, the effects of defective central fractalkine-CX3CR1 signalling.

The present study demonstrated that fractalkine-CX3CR1 signalling has anorexigenic and anti-inflammatory actions in the hypothalamus of diet-induced obese mice. Of considerable interest for the present study is the possibility that CX3CR1 activation may be a promising way to reduce body weight and improve brain inflammation associated with obesity. Our study provides that fractalkine-CX3CR1 signalling may be an attractive target for the pharmacological treatment of obesity.

## Methods

### Animals

Male C57BL/6 J mice (6 weeks old) were obtained from CLEA Japan, Inc. (Tokyo, Japan). CX3CR1-deficient mice, in which both copies of CX3CR1 were disrupted by the gene for enhanced green fluorescent protein^52^, were initially obtained from The Jackson Laboratory and then bred to maintain them on the C57BL/6 J background.

Mice were housed in plastic cages in a room kept at room temperature of 23 ± 1 ℃ with a 12: 12 h light–dark cycle (lights turned on at 7:00 _A.M._). Ad libitum access to water and food (CE-2; CLEA Japan, Inc.) was provided to all mice. The mice were used in the experiments at 6–16 weeks of age. All animal experiments complied with the ARRIVE guidelines and were performed in accordance with the guidelines established by the Institute of Laboratory Animal Science Research Support Center at Kagoshima University and approved by the Kagoshima University Institutional Animal Care and Use Committee (protocol nos. MD15060, MD16052、MD17060, and MD18079), and in accordance with the guidelines established by the United States National Institutes of Health Guide for the Care and Use of Laboratory Animals (NIH publication No. 80-23, revised in 1996). Every effort was made to minimize the number of animals used and to optimize their comfort.

### Peptides and drugs

Fractalkine was purchased from Wako Pure Chemical Industries, Ltd. (Osaka, Japan). Ghrelin and NPY were purchased from Peptide Institute, Inc. (Osaka, Japan). BDNF was purchased from PeproTech, Inc. (Rocky Hill, NJ). ANA-12 was purchased from Merck KGaA (Darmstadt, Germany).

### Intracerebroventricular injection

Intracerebroventricular injection was performed according to our previous report^53^. The coordinates for intracerebroventricular injection were 1.8 mm lateral from the midsagittal suture, 0.7 mm posterior to the bregma, and − 2.5 mm from the flat skull surface.

### Fasting-refeeding

Male mice at 10–12 weeks of age were used in the experiments. Mice were fasted for 48 h and then refed for 4 h. Water was available ad libitum during the experiments. Fractalkine was dissolved in saline. Intracerebroventricular administration of fractalkine (1, 3, 10 μg/2 μl/mouse) was performed just before refeeding. Mice in the control group were given an intracerebroventricular administration of an equal volume of saline. Food intake was measured during 4 h of refeeding. At the end of the experiments, the hypothalamus was collected for examination of the mRNA expression for neuropeptides and their receptors^54^.

### Nocturnal food intake

Male mice at 10–12 weeks of age were used in the experiments. To assess the effect of intracerebroventricular administration of fractalkine on nocturnal food intake, fractalkine (3 μg/2 μl/mouse) was injected intracerebroventricularly 1 h before the beginning of the dark phase. Mice in the control group were given intracerebroventricular administration of an equal volume of saline. Food intake was measured for 15 h after intracerebroventricular injection. Food and water were available ad libitum at all times.

### Food intake induced by NPY and ghrelin

Male mice at 10–12 weeks of age were used in the experiments. The experiments were performed from 11:00 _A.M._ to 3:00 _P.M._ Fractalkine (3 μg/2 μl/mouse) was intracerebroventricularly administered just before intracerebroventricular administration of NPY (20 μg/2 μl/mouse; dissolved in saline) or intraperitoneal administration of ghrelin (360 μg/kg, 10 ml/kg body weight; dissolved in saline). Mice in the control group were given an intracerebroventricular or intraperitoneal administration of an equal volume of saline. Food intake was measured for 4 h after peptide injection. Food and water were available ad libitum at all times.

### Intraperitoneal administration of ANA-12

ANA-12 was dissolved in dimethyl sulfoxide (DMSO), and diluted with saline in a final concentration of 2% DMSO. Male mice at 10–12 weeks of age were used in the experiments. ANA-12 (0.5 mg/kg, 10 ml/kg body weight) was intraperitoneally administered after 48-h fasting. In the vehicle treatment groups, an equal volume of vehicle (2% DMSO saline) was intraperitoneally administered. Fractalkine (3 μg/2 μl/mouse) and BDNF (0.5 μg/2 μl/mouse) were intracerebroventricularly administered 30 min after the administration of ANA-12 and then food intake was measured for 4 h after peptide injection. Food and water were available ad libitum during the experiments.

### Food intake of CX3CR1-deficient mice

Male CX3CR1-deficient mice at 10–12 weeks of age were used in the experiments. Cumulative food intake was measured for 3 days. Food and water were available ad libitum during the experiments. At the end of experiments, the hypothalamus was collected to examine BDNF mRNA expression under isoflurane anesthesia^54^.

### Analysis in mice fed a HFD

Normal male mice (6 weeks old) were randomly divided into two groups. The first group was given CE-2 as a CD containing 12.6% fat of total calories (343.1 kcal/100 g). The second group was given a HFD (no. D12492; Research Diets, Inc., New Brunswick, NJ) containing 60% fat of total calories, predominantly in the form of lard (524 kcal/100 g). After feeding on the diets for 2, 4, 8, or 16 weeks, the hypothalamus was collected under isoflurane anesthesia.

Fractalkine was dissolved in saline. The mice fed a HFD for 16 weeks were killed at 3 h after the intracerebroventricular administration of fractalkine (3 μg/mouse), and the hypothalamus was collected under isoflurane anesthesia. In the control group, an equal volume of saline (2 μl/mouse) was intracerebroventricularly administered.

Male CX3CR1-deficient mice (14–16 weeks old) were randomly divided into two groups. The first group was given a CD. The second group was given a HFD. After feeding on the diets for 2 or 4 weeks, the hypothalamus, mesenteric fat, and epididymal fat were collected under isoflurane anesthesia.

### Sampling of the hypothalamus

Mice were killed by decapitation. The hypothalamus was rapidly removed from the skull and placed on an ice-cooled paraffin plate for dissection of the hypothalamus as previously described^54^. The hypothalamus was immediately frozen in liquid nitrogen and stored at − 80 °C until analyzed.

### MG6 microglial cell culture

MG6 cells, a mouse microglia cell line (RCB 2403, RIKEN Cell Bank, Tsukuba, Japan) were maintained in a growth medium composed of Dulbecco’s modified Eagle’s medium (DMEM) containing 10% fetal bovine serum supplemented with 100 μM β-mercaptoethanol, 10 μg/ml insulin, 100 μg/ml streptomycin and 100 U/ml penicillin in 90-mm Petri dishes (Thermo Fisher Scientific Inc., Waltham, MA) at 37 °C in 5% CO_2_ and 95% air^55,56^. The MG6 cells were replated in 6-well plates at a density of 1 × 10^5^ cells/ml in normal DMEM medium. After 4 days, MG6 cells were used in the experiment. To examine the effects of fractalkine on BDNF mRNA expression, fractalkine (100 nM) was applied to the MG6 cells for 3 h. The treated cells were collected with Lysis RLT buffer (QIAGEN, Venlo, Netherlands) containing 1% β-mercaptoethanol for analysis using real-time reverse transcription polymerase chain reaction (RT-PCR).

### RT-PCR

The extraction of total RNA from mouse hypothalamus and MG6 cells was performed according to our previous report^57^. cDNA was synthesized from total RNA samples using a Verso cDNA Synthesis Kit (Thermo Fisher Scientific Inc.). Quantitative real-time RT-PCR was performed using FastStart SYBR Green Master (Roche Applied Science, Basal, CHE) on a thermal Cycler Dice Real Time System (TAKARA BIO INC., Shiga, Japan) according to our previous report^57^. All gene-specific mRNA expression values were normalized against the internal housekeeping gene, glyceraldehyde-3-phosphate dehydrogenase (GAPDH). Primer sets are shown in Table 1.

**Table 1:** Primers used for RT-PCR.

Genes	Oligonucleotides sequences	
	Forward	Reverse
GAPDH	TGCACCACCAACTGCTTAGC	GGATGCAGGGATGATGTTCTG
Fractalkine	ACGAAATGCGAAATCATGTGC	CTGTGTCGTCTCCAGGACAA
CX3CR1	CGTGAGACTGGGTGAGTGAC	AAGGAGGTGGACATGGTGAG
NPY	CTGACCCTCGCTCTATCTCTG	AGTATCTGGCCATGTCCTCTG
Y1	AACCTCTCCTTCTCAGACTT	GTAAGACAGCCTGTGAGAGT
Y5	GAGAAGCACCTAACCGTTCCAG	TGAGGGAACGCTTGACTCTCAT
AgRP	TTGTGTTCTGCTGTTGGCACT	AGCAAAAGGCATTGAAGAAGC
POMC	CCCAACGTTGCTGAGAACGAGTCG	GGAGGTCATGAAGCCACCGTAACG
MC3R	CATTGCCATCGACAGGTACGT	CTGTGGTACCGAAGGGCATAG
MC4R	AGCCTGGCTGTGGCAGATAT	GGTTTCCGACCCATTCGAA
BDNF	TGCAGGGGCATAGACAAAAGG	CTTATGAATCGCCAGCCAATTCTC
TrkB	CCGGCTTAAAGTTTGTGGCTTAC	GGATCAGGTCAGACAAAGTCAAG
Orexin	GCCGTCTCTACGAACTGTTGC	CGCTTTCCCAGAGTCAGGATA
MCH	ATTCAAAGAACACAGGCTCCAAAC	CGGATCCTTTCAGAGCAAGGTA
CART	AGAGTAAACGCATTCCGATCTACGA	TCCTCACTGCGCACTGCTCT
TNF-α	CTGTGAAGGGAATGGGTGTT	GGTCACTGTCCCAGCATCTT
IL-1β	CTCCATGAGCTTTGTACAAGG	TGCTGATGTACCAGTTGGGG
IL-6	TCCAGTTGCCTTCTTGGGAC	GTGTAATTAAGCCTCCGACTTG

*GAPDH* Glyceraldehyde-3-phosphate dehydrogenase, *NPY* Neuropeptide Y, *AgRP* Agouti-related protein, *POMC* Proopiomelanocortin, *MC3R* Melanocortin-3, *MC4R* Melanocortin-4 receptors, *BDNF* Brain-derived neurotrophic factor, *MCH* Melanin-concentrating hormone, *CART* Cocaine- and amphetamine-regulated transcript peptide, *TNF-α* Tumour necrosis factor-α, *IL-1β* Interleukin-1β, *IL-6* Interleukin-6.

### Data analysis

All values are given as the mean ± SEM. Statistical analysis of the data was performed by ANOVA, followed by the Tukey–Kramer test. Statistical significance was defined as *P* < 0.05.

## Data Availability

The datasets generated during and/or analyzed during the current study are available from the corresponding author on reasonable request.
